# The E3 ligase UBR2 regulates cell death under caspase deficiency via Erk/MAPK pathway

**DOI:** 10.1038/s41419-020-03258-3

**Published:** 2020-12-08

**Authors:** Elodie Villa, Rachel Paul, Ophélie Meynet, Sophie Volturo, Guillaume Pinna, Jean-Ehrland Ricci

**Affiliations:** 1 Université Côte d’Azur, INSERM, C3M Nice, France; 2 Université Paris-Saclay, CEA, CNRS, Institute for Integrative Biology of the Cell (I2BC), 91198 Gif-sur-Yvette, France

**Keywords:** Cancer, Cell death

## Abstract

Escape from cell death is a key event in cancer establishment/progression. While apoptosis is often considered as the main cell death pathway, upon caspase inhibition, cell death is rather delayed than blocked leading to caspase-independent cell death (CICD). Although described for years, CICD’s underlying mechanism remains to be identified. Here, we performed a genome-wide siRNA lethality screening and identified the RING-Type E3 Ubiquitin Transferase (UBR2) as a specific regulator of CICD. Strikingly, UBR2 downregulation sensitized cells towards CICD while its overexpression was protective. We established that UBR2-dependent protection from CICD was mediated by the MAPK/Erk pathway. We then observed that UBR2 is overexpressed in several cancers, especially in breast cancers and contributes to CICD resistance. Therefore, our work defines UBR2 as a novel regulator of CICD, found overexpressed in cancer cells, suggesting that its targeting may represent an innovative way to kill tumor cells.

## Introduction

The process of cell death affects essentially all cell types in multicellular animals. Programmed cell death (PCD) regroups a wide range of molecular mechanisms showing considerable overlapping^1^. It is often considered that most programmed cell death in animals occur by apoptosis and most apoptosis in mammals occurs through the mitochondrial pathway. In the simplest description of the mitochondrial pathway, signals that elicit apoptosis (e.g., DNA damage, loss of adhesion, withdrawal of survival factors, and others) activate pro-apoptotic members of the Bcl-2 family, in particular Bax and Bak, to form pores in the outer membrane of the mitochondria. Anti-apoptotic members of the Bcl-2 family prevent this event, and thereby block apoptosis^2^. The mitochondrial outer membrane permeabilization (MOMP) allows proteins in the intermembrane space to diffuse out of the mitochondria and interact with proteins in the cytosol. One central player is cytochrome c, which once released from the mitochondria interacts with Apaf-1. This interaction will result in the oligomerisation of Apaf-1 and recruitment of caspase-9 forming therefore a large structure called the apoptosome. Caspase-9 is inactive in its monomeric state, but upon binding to oligomerized Apaf-1, the caspase becomes active and cleaves its substrates, the executioner caspases-3 and −7. This cleavage event activates the executioner caspases, which then orchestrate apoptosis by cleaving specific substrates within the cell^3^.

Within a short time of identifying caspases as the enzymes that orchestrate apoptotic cell death, it became apparent that inhibition of caspase activity may not necessarily preserve cell survival even if the features of apoptosis are effectively blocked^4^. Although initially viewed as a passive or default pathway, accumulating evidence suggests that at least some forms of non-apoptotic form of death are programmed and regulated^5^.

Among those, caspase-independent cell death” (CICD) is defined as a cell death occurring post-MOMP, in conditions where caspases are not activated. Importantly CICD can be blocked upon Bcl2 overexpression, which prevents MOMP, but can proceed in cells lacking Apaf-1^6,7^ (cells that are unable to engage caspase activation as a consequence of cytochrome c release). CICD often shares common characteristics with apoptotic cell death, such as MOMP and not swollen mitochondria. However, typical characteristics of apoptosis (such as phosphatidyl serine externalization and wide-scale chromatin condensation) are often not observed upon CICD. Instead, cells dying by CICD will present large-scale cytoplasmic vacuolization, autophagosome accumulation, and peripheral nuclear condensation. Compared to apoptosis, CICD is a slow, albeit very effective, cell death process^7^. The existence of CICD is supported by numerous findings in both cell culture models and in vivo studies using various mice models deficient in apoptotic signaling (for review^7^). Recently, it was suggested that CICD may also present potent anti-tumorigenic effects, through the induction of an efficient anti-cancer immune response^8^. However, a precise molecular definition of this non-apoptotic form of PCD still remains to be found. In fact, a lack of a robust and easy way to investigate CICD in cell culture as in vivo is currently hampering our research capability concerning the study and the modulation of this form of cell death.

In this study, we set up a genome-wide siRNA screen to identify novel regulators of caspase-independent cell death.

## Materials/subjects and methods

### Cell culture

HeLa, MDAMB231, MCF7, T47D, and BT474 where obtained from ATCC cells were cultivated in Dulbecco’s minimum essential medium supplemented with 10% fetal bovine serum. Cells were maintained in 5% CO_2_ at 37 °C. HeLa SMAC-GFP, 3T3-SA were a kind gift of Dr. S.W. Tait.

### Identification of genes modulators of CICD by High Content Screening

We performed a systematic, individual, and transient gene loss-of-function screening to identify genes regulating the Caspase-Independent Cell Death of a HeLa cell line engineered to stably and constitutively express an shRNA against hAPAF1. To achieve this, we used a human genome-wide siRNA library constituted of individual siRNAs (3 siRNAs/target gene) arrayed in 384-well format and designed to specifically target and knockdown 22,950 human genes (human whole genome-wide library V1.0, Qiagen). For screening purpose, an automated forward transfection protocol was developed on a robotic workstation equipped with a 96-well head probe (Nimbus, Hamilton). Briefly, HeLa-shAPAF1 cells were labeled for 30 min with the vital fluorescent dye 5-chloromethylfluorescein diacetate (Cell Tracker Green CMFDA, Cat# C925, Sigma) directly into the culture flask (final [CMFDA] = 5 µM). Cells were then trypsinized and seeded into the wells of a clear bottom, black-walled 384-well culture plates (ViewPlates 384, Perkin Elmer, Cat# 6007460) at a density of 3000 cells/well. After 3 h of incubation (37 °C and 5% CO_2_) to allow for cell attachment, individual siRNAs were complexed with Lipofectamine RNAiMAX (Life Technologies) for 15 min, and the resulting lipoplexes were layered on top of the culture wells (final [siRNA] = 25 nM). To minimize positional errors, each individual siRNA from the library was transfected as a separate duplicate in different well positions of two independent culture plates. Each culture plate also received different positive and negative controls: 12 wells received the transfection reagent alone (“MOCK” well, negative controls), 28 were transfected with a scrambled siRNA sequence (“UNR” Wells, custom target sequence: AAGCCGGTATGCCGGTTAAGT, Qiagen), and 8 were transfected with a pool of cytotoxic siRNAs (“AllStars Death” wells, positive control, Allstars maximal death control, Qiagen). 24 h post-transfection, cell death was induced by adding 10 µL of a suspension of Actinomycin D (Sigma, Cat# A9415) to the culture wells (Final [Act.D] = 100 nM). 96 h post-transfection, cells were fixed by formalin at 4% (w/v) final (Cat# 47608, Sigma) and nuclear DNA was fluorescently labeled with Hoechst 33342 (2 µg/mL, Cat# B2261, Sigma). Plates were imaged on a high content imaging microscope (Operetta, Perkin Elmer) at 10X magnification (4 fields/well), in two fluorescence channels: green for CMFDA (ex: 470 ± 10 nm; em: 525 ± 25 nm) and blue for Hoechst 33342 (ex: 380 ± 20 nm; em: 445 ± 35 nm). An automated algorithm was developed under Harmony 3.0 (Perkin Elmer) to quantify the amount of dying cells upon gene knockdown. Briefly, nuclear and cytoplasmic regions of interest (ROI) were segmented in the Hoechst and CMFDA channels, respectively. Nuclear ROIs were then cleaned to remove truncated nuclei events located on the edges of the images and other small debris. Nuclear roundness (R), nuclear contrast (C), and cytoplasmic area (A) were then computed from the nuclear and cytoplasmic ROIs; with (R) and (C) being scores, comprised between 0 and 1, reflecting how much a given ROI is close to a perfect circle (R = 1 being a perfect circle, R = 0 a random shape), or how far the average ROI fluorescence deviates from its surrounding background (C = 1 being an infinite contrast, C = 0 indicating no difference from the background). Macroscopically dead/dying cells were separated from healthy cells by setting up R.C and S thresholds in the maximal death control wells. unhealthy cells were selected when R.C > 0.5 and S < 414.4 µm^2^ (2 SD below the average Cytoplasmic ROI surface of dead/dying cells in the maximal death control wells). The secondary screen focused only on the subset of genes selected as candidates, retested within a similar setup with 4 siRNAs/gene (the 3 same siRNAs as in the primary screen + 1 another individual siRNA targeting the same gene). For each culture plate, the percentage of unhealthy cells measured in sample wells were first normalized to those measured in their respective negative control (UNR) wells.

### Genome wide siRNA lethality screening

When less than 30% of cell death was observed with a siRNA, the gene was considered as a sensitizer gene. When more than 70% of cell death was observed with a siRNA, the gene was considered as a protector gene.

For the network and pathway analysis of CICD screen results the HPRD (Human Protein Reference Database) -curated genes from a combined set of Low confidence set of genes and High confidence set of genes (**ALL confidence set of genes**: 519 HPRD-curated genes from 1307 genes total) were used. The analysis was performed using Cytoscape Software and BiNom, Bingo, and ClueGO plug-ins for it.

### Transfections and gene silencing

For plasmid transfections, HeLa cells were seeded at 1 × 10^5^ cells/well in 6-well plates 24 h prior to transfection. HeLa were transfected according to the standard calcium phosphate precipitation method.

HeLa shApaf-1 was obtained using the SureSilencing shRNA Plasmid for Human APAF1 (KH00752P).

For siRNA transfections, HeLa cells (2 × 10^5^ cells/well) were transfected with Lipofectamine RNAimax (ThermoFisher) following manufacturer’s instructions.

siRNAs were used: control (non-targeting) siRNA (GAUUUAUGCAACAAUAGUA), siUBR2 #1 (GCGTTTGCAGAGTGATTATGT), siUBR2#2 (CCAATGGAATGGTACCTTT), siUBR2#3 (GCTGCTTCCTCCAAGAAAT), si mouse UBR2 #1(GCAAGTCATTTATACCCTT), si mouse UBR2#2 (CCAGAGCTCCTACCTCTAA), siUBE2V2#1 (GGTGGACAGGCATGATTAT), siUBE2V2#2 (GACGTCTAATGATGTCCAA), siEDD (GCAGTGTTCCTGCCTTCTT), siITCH (GGAGCAACATCTGGATTAATA), siARIH1#1 (CCACTTCAATTGGGATAAA) and siARIH1#2 (CCAATATCCTGATGCTAAA). siPSMB3 (SASI_Hs01_00216057) were purchased from Sigma Aldrich.

UBR2-his-tagged (PV453137) was purchased from ABM.

### Cell death measurement

To induce cell death, cells were treated either with Actinomycin D, Staurosporine, Mitomycine D, TNF-α, Erastin or irradiated with an UV lamp (254 nm) with indicated doses. Cell viability of the treated cells was assessed by looking at the plasma membrane permeabilization using either a DAPI or a Propidium Iodide staining and then analyzed by flow cytometry (Miltenyi Biotec) or using the Incucyte system. For the kinetic of cell death, cells were seeded in 48-well plates and incubated for the indicated. Cell confluence was imaged by phase-contrast and red fluorescence using the IncuCyte HD system (IncuCyte™ live-cell). Frames were captured at 1 h intervals from 2 separate regions/well using a × 10 objective. Cell death curves were constructed by imaging plates using IncuCyte™ Zoom software.

To assess DEVDase activity, the cells were lysed in buffer (Hepes 10 mM pH 7.4, NaCl 150 mM, EDTA 5 mM, 1% NP40, 10 μg/ml aprotinin, 1 mM PMSF, 10 μM leupeptin) 16 h after treatment. Lysates were standardized for protein content and loaded into a black 96-well plate (CellStar) in the presence of 0.2 mmol/L of the caspase-3 substrate Ac-DEVD-AMC diluted in the following buffer: 50 mmol/L HEPES (pH 7.5), 150 mmol/L NaCl, 20 mmol/L EDTA, and 10 mmol/L DTT. Caspase activity was determined both with and without the presence of 1 μmol/L of the Caspase 3 inhibitor Ac-DEVD-CHO using a fluoroscan at 460 nm, and the specific activity was expressed as the change in absorbance per minute per milligram of protein.

To assess Apoptosome formation, cell lysates were prepared by resuspending cells for 20 min at 4 °C in buffer A (Hepes 10 mM pH 7.4, NaCl 150 mM, EDTA 5 mM, 1% NP40, 10 μg/ml aprotinin, 1 mM PMSF, 10 μM leupeptin) followed by centrifugation at 10,000 g for 20 min. Lysates were standardized for protein content, incubated with 1 μM bovine heart cytochrome c (Sigma St Louis MI) and 1 mM ATP (Fermentas Thermo Fisher Rockford IL) at 37 °C and loaded into a black 96-well plate (CellStar) in the presence of 0.2 mmol/L of the caspase-3 substrate Ac-DEVD-AMC diluted in the following buffer: 50 mmol/L HEPES (pH 7.5), 150 mmol/L NaCl, 20 mmol/L EDTA and 10 mmol/L DTT. Caspase activity was determined both with and without the presence of 1 μmol/L of the Caspase 3 inhibitor Ac-DEVD-CHO using a fluoroscan at 460 nm, and the specific activity was expressed as the change in absorbance per minute per milligram of protein.

### Clonogenic assays

HeLa cells were seeded at 2 × 10^4^ cells/well in 12-well plates and treated 6 h with Actinomycin D and qVD-OPH. After media was refreshed. Once clone formation was observed, cells were stained with crystal violet (Sigma Aldrich).

### Western Blot

After treatment, the cells were collected, washed in PBS, and lysed in lysis buffer (SDS 20%, TRIS 0.5 M pH 6.8, Glycerol 10 %) Proteins (50 μg) were separated on polyacrylamide gels (10% and 15%) and transferred onto PVDF membranes. After the nonspecific binding sites had been blocked, the membrane was incubated overnight at 4 °C with the primary antibody. The membrane was washed 3 times with a buffer containing Tris 50 mM, NaCl 150 mM pH 7.5, and 1% NP40 and incubated with the HRP-conjugated secondary antibody for 1 h at room temperature. Immunoblots were visualized using the enhanced chemiluminescence detection kit (Pierce).

### Reagents and antibodies

Antibodies for western-blot were obtained from the following suppliers: anti-PSMB3 (D-17), anti-ITCH (D-20), anti-EDD (M-19), anti-LAMP2 (H4B4), anti-actin (C-4), and anti-ERK2 (D-2) were from Santa-Cruz Biotechnology. Anti-UBE2V2 (PAB6156) was purchased from Abnova. Anti-UBR2 (18853-1-AP) was purchased from Euromedex. Anti Apaf-1 (#8969), anti-Mcl1 (#94296) anti-cytochrome c (D18C7), anti-Smac (D5S3R), and Phospho-Erk1/2 Pathway Sampler Kit (#9911) were purchased from Cell Signaling Technologies.

Actinomycin D, Staurosporine, Mitomycin C, and Erastin were obtained from Sigma-Aldrich. Velcade (Bortezomib) was obtained from Calbiochem. qVD-OPH and zVAD.fmk were obtained from SM Biochemicals LLC. ABT-737 was purchased from Sigma.

### Bio informatic analysis of clinical data

The data were generated in^9^. Fifty-three breast tumor stroma samples and six normal breast stroma samples were analyzed on Agilent 44 K microarrays. The data in Oncomine has been processed by inversing the ratios and averaging the two values per sample.

Patient survival curves were obtained using DrugSURV by analyzing the GEO dataset ID GSE24450 that contains “183 breast tumors from the Helsinki University center hospital with survival information”. UBR2 (ID 23304, probe ID: ILMN_1663489) levels were defined compared to the median UBR2 expression in all patients.

### Statistical Methods

Data are expressed as the means ± standard deviation (SD). Differences in the calculated means between the groups were assessed by two-way ANOVA test.

## Results

### Genome-wide screening to identify CICD regulators

**Fig. 1:**
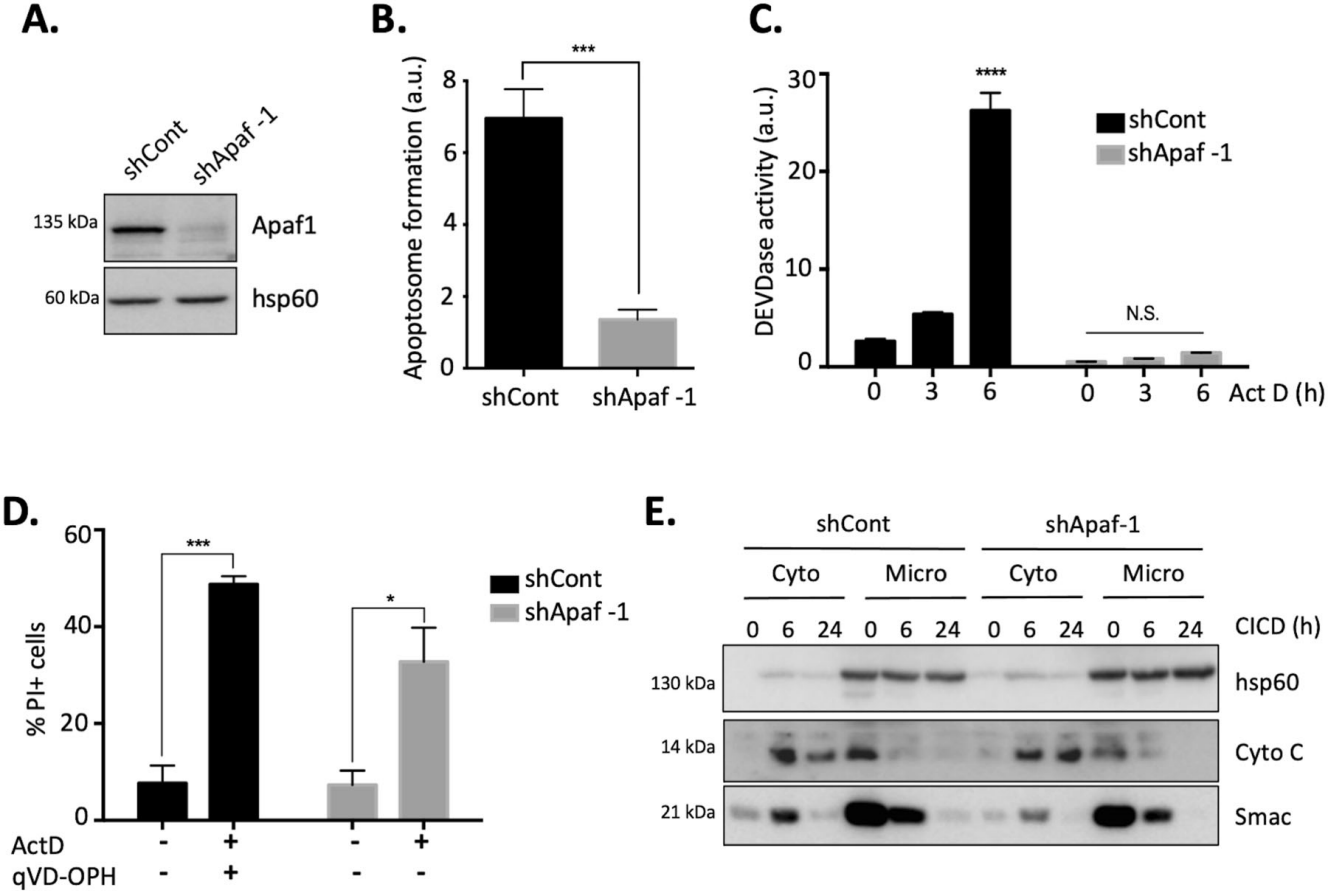
Generation of a cell line model to identify CICD regulators. HeLa cells were stably transfected with either a non-targeting shRNA (shCont) or a shRNA targeting Apaf-1 (shApaf-1). **A** immunoblotting for Apaf-1 in whole cell lysates isolated from both cell types (hsp60 was used as a loading control), **B** protein extracts were isolated from HeLa shCont or shApaf-1 cells and apoptosome formation was measured in vitro upon addition of cytochrome c, **C** HeLa shCont or shApaf-1 cells were treated with Actinomycin D (1 µM, as an apoptosis induction) for the indicated times and DEVDase activity was measured. **D** HeLa shCont were treated for 3 days with Act D (1 µM, to induce apoptosis) or with Act D (1 µM) + qVD-OPH (20 µM) to induce CICD. HeLa shApaf-1 cells were treated for 3 days with Act D (1 µM). qVD-OPH was not added to HeLa shApaf-1 cells as those cells cannot activate caspases upon Act D treatment (see **B** and **C**). Cell death was analyzed by flow cytometry using a Propidium Iodide (PI) staining. **E** HeLa shCont or shApaf-1 cells were treated with Actinomycin D + qVD-OPH (1 μM or 20 μM respectively) as a CICD stimulus for the indicated times. Isolation of cytosolic (cyto) and microsomal fractions (micro, which contains mitochondria in particular) was assessed by cell fractionation. MOMP, assessed by looking at the relocalization of Cytochrome c (Cyto C) and Smac from the mitochondria to the cytosol upon CICD treatment, was measured by immunoblotting. hsp60 was used as a control for the presence of mitochondria in the microsomal fraction. Data are expressed as mean ± s.d (*n* = 3), immunoblots are representative of 3 or more individual experiments. *p < 0.05, **p < 0.01, ***p < 0.001, ****p < 0.0001 according to a two-way ANOVA. N.S: non-significant.

As CICD occurs post-MOMP and upon caspase deficiency, we first generated HeLa cells stably knocked down for Apaf-1 (HeLa-Apaf-1 KD) to identify the molecular actors participating to CICD regulation (Fig. 1A). Apaf-1 silencing was chosen as it was shown in several studies, including in vivo models, to completely prevent apoptosis but not CICD occurrence^6,10^. Adding cytochrome c to a protein cell extract will allow in vitro apoptosome formation (composed of Apaf-1, caspase 9, and cytochrome c) and subsequent caspase activation can be measured using a caspase −3/−7 colorimetric specific peptide (DEVDase activity). We verified that HeLa-Apaf-1 KD was indeed unable to assemble apoptosome and therefore unable to activate caspases in vitro in presence of cytochrome c (Fig. 1B), confirming that upon MOMP, HeLa-Apaf-1 KD cells will not be able to die by apoptosis. Actinomycin D (Act D, a DNA to RNA transcription inhibitor) is a typical inducer of cell death in HeLa cells. It will induce MOMP followed by caspase-dependent apoptosis within hours (typically from 2 to 6 h) and it will induce CICD if caspases cannot be activated (typically within 2 to 3 days)^7,11^. We confirmed that HeLa-Apaf-1 KD was not able to form the apoptosome and then to activate caspases upon Act D stimulation (Fig. 1C), while caspase activation was obvious in control cells.

We then determined if Apaf-1 KD was able to prevent apoptosis but not CICD as expected. We compared Act-D-induced CICD in control cells (by combining Act D with the covalent pan-caspase inhibitor q-VD-OPH–quinolyl-valyl-O- methylaspartyl-[2,6-difluorophenoxy]-methyl ketone) to the CICD obtained in HeLa-Apaf-1 KD upon treatment with Act D alone (Fig. 1D). qVD-OPH was not added to HeLa-Apaf-1 KD, as they cannot activate caspases (Fig. 1C) and are therefore unable to die by apoptosis. We observed that upon treatment with Act D alone, HeLa-Apaf-1 KD were dying by CICD to a similar extent than control cells cotreated with Act-D and qVD-OPH (Fig. 1D).

CICD is defined as occurring post-MOMP, and because preventing MOMP will preclude apoptosis and CICD^7^, it was, therefore, important to validate that MOMP was not altered in HeLa-Apaf-1 KD cells. For that matter, we measured cytochrome c and Smac release from the mitochondria. Once release from the mitochondria (microsomal fraction in Fig. 1E) cytochrome c will accumulate in the cytosolic fraction. Smac, upon its release from the mitochondria, will be rapidly targeted for degradation^12^. While Smac was observed in the microsomal fraction in control and HeLa-Apaf-1 KD cells in the absence of stimuli, it was degraded to a similar extent and with a similar kinetic in both cells upon induction of CICD (Fig. 1E).

We concluded that MOMP was not affected in HeLa-Apaf-1 KD compared to control cells and that HeLa-Apaf-1 KD cells represent a suitable model to identify CICD regulators.

**Fig. 2:**
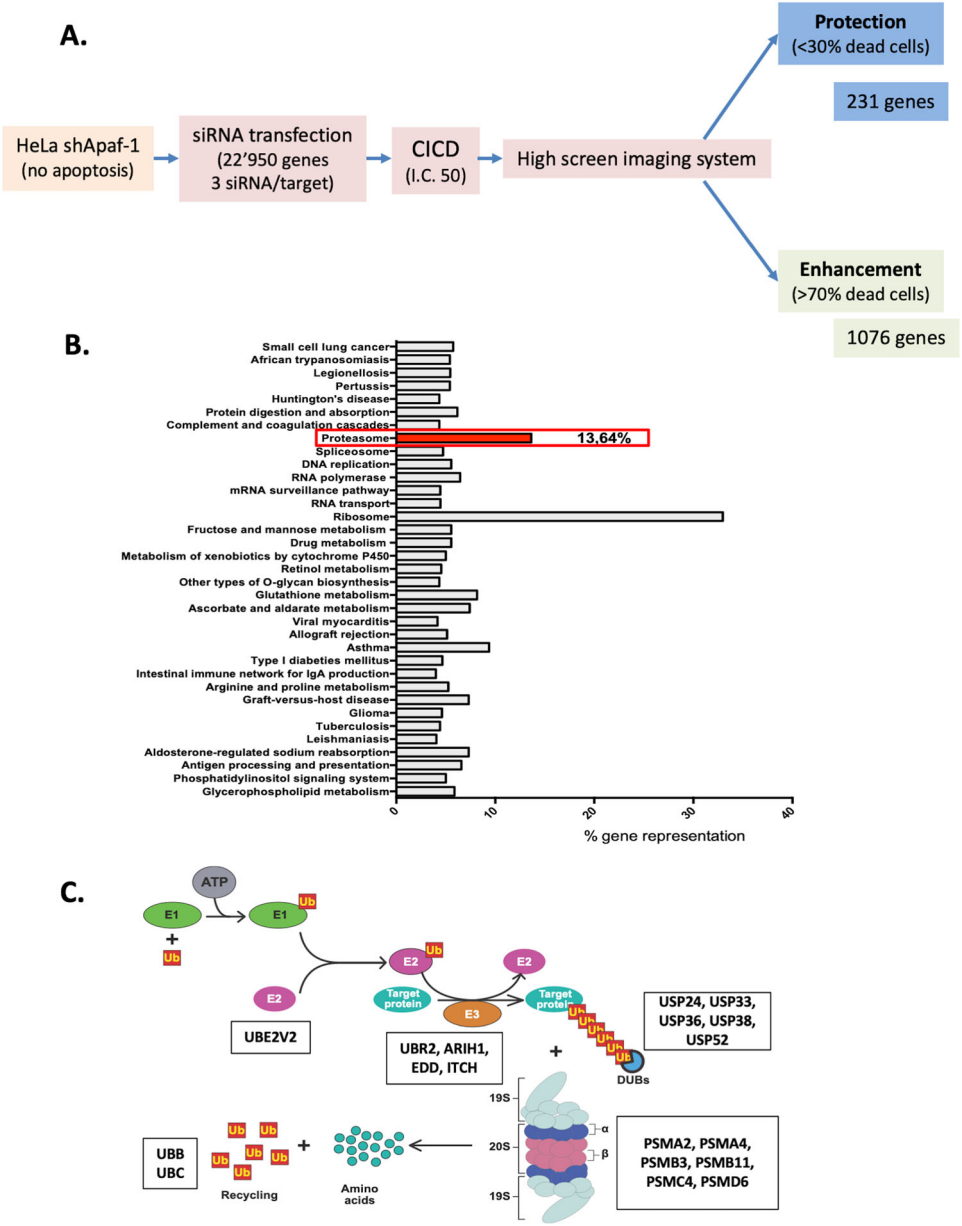
Genome wide screening to identify CICD regulators. **A** summary of the protocol for the genome wide siRNA lethality screening on HeLa Apaf-1 KD cells and identification of CICD regulators. **B** Distribution of all the enriched pathways found for the genes in the hit list. Genes were grouped according to their participation in the indicated processes. KEGG Pathway database was used, altogether with a combination of several plug-ins in Cytoscape software and a set of appropriate statistical analysis. **C** Schematic representation of the Ubiquitin-Proteasome System (UPS). All the UPS genes identified as regulators in the CICD screening are indicated in boxes.

Using the Apaf-1 KD model, we performed a genome-wide siRNA lethality screen covering the human genome (22’950 genes, Fig. 2A). These apoptotic deficient cells were transfected by individual siRNAs (3 siRNAs/target gene, each tested in duplicates) and CICD was induced with Act D. Changes in cellular and of nuclear morphologies occurring in the early stages of cell death were detected and quantitated by high content imaging (Figure S1). Briefly, we computed a morphology filtering algorithm based on cellular morphology and nuclear shrinkage that allowed the separation of dying from healthy cells. For screening purpose, Actinomycin D concentration was adjusted so that approximately 50% of cells presented a “dying” phenotype. The genes whose silencing induced the most dramatic morphological changes compared to the control conditions were selected as potential CICD modulators. Using this approach, we identified 231 (1% of 23,079 tested genes) and 1076 genes (4.7%) whose extinction led to potential CICD protection (i.e., <30% dead cells upon silencing, called sensitizing genes) or enhancement (i.e., >70% dead cells, called protective genes), respectively (see supplementary table 1 for the list of identified genes and supplementary Fig. 1B for a GO analysis of the results).

Bio-informatics analysis of the signaling pathways enriched by these genes are indicated in Fig. 2B. One pathway stood out in particular, the Ubiquitin- Proteasome System (UPS). The UPS is a key player in protein homeostasis and cell signaling as it is the main process by which proteins are degraded^13^. 18 genes of the UPS were identified as potential CICD regulators. These genes intervene in almost every step of this process (Fig. 2C).

### Knockdown of UBR2 sensitizes cells to CICD but not to apoptosis

**Fig. 3:**
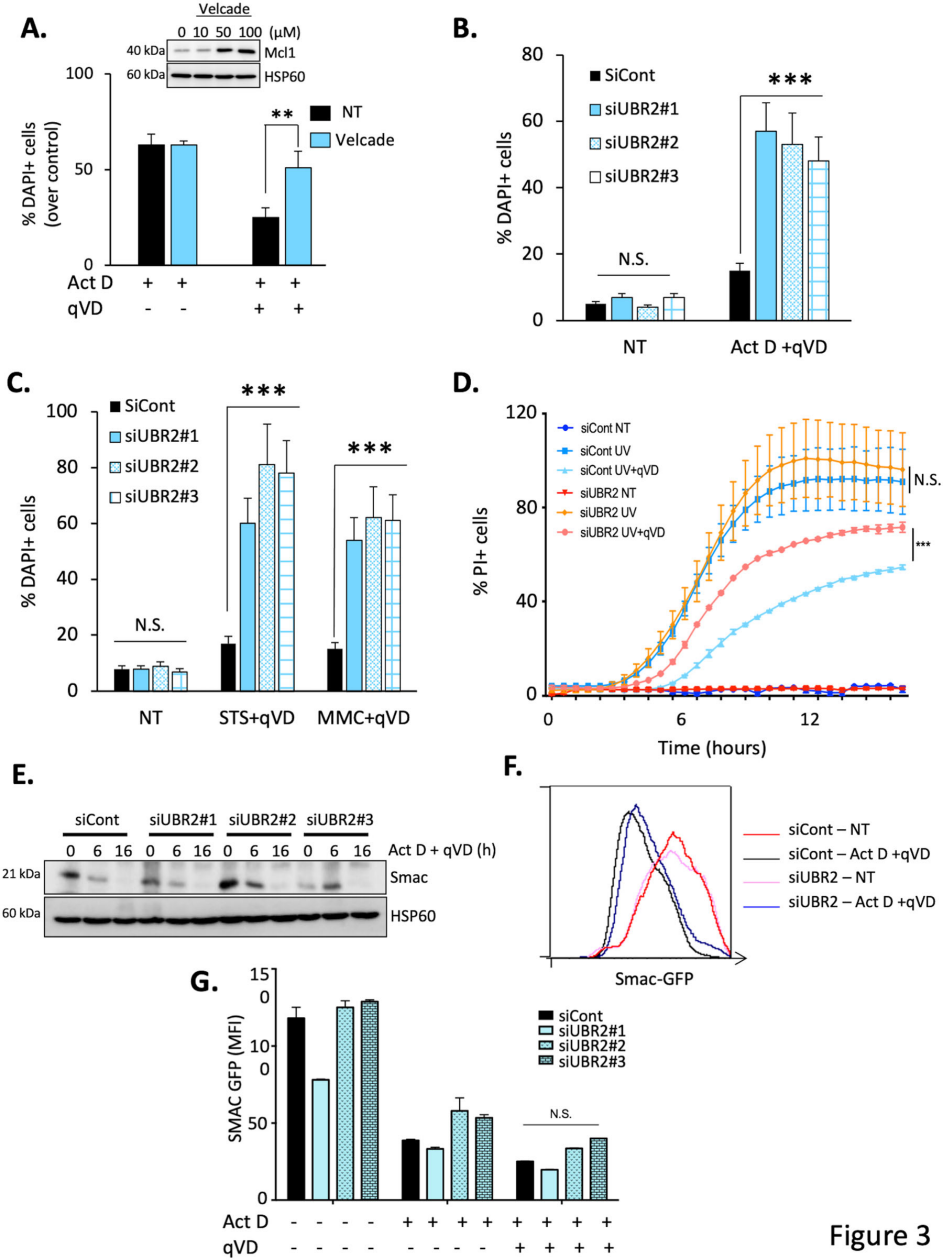
UBR2 knockdown sensitizes cells to CICD. **A** HeLa cells were treated with Actinomycin D (1 µM) alone (to induce apoptosis) or in combination with the proteasome inhibitor Velcade (50 μM) for 8 h and cell death was measured by flow cytometry using a Propidium Iodide staining. In parallel, HeLa cells were treated with Actinomycin D (1 µM) and qVD-OPH (20 μM) alone or in combination with Velcade (50 μM) for 36 h and cell death was measured by flow cytometry using a Propidium Iodide staining. The results are expressed as cell death measured over control condition. Velcade efficiency was assessed by looking at the stabilization of Mcl-1 by immunoblotting. Hsp60 was used as a loading control. HeLa cells were transfected either with a non-targeting siRNA (siCont) or with three different siRNAs (20 nM) targeting UBR2 (siUBR2). 48 h post-transfection **B** control cells or cells knocked down for UBR2 were either not treated (NT) or treated with Actinomycin D (1 μM) in combination with the pan-caspase inhibitor qVD-OPH (20 μM) as a « CICD » stimulus for 36 h and cell death was analyzed by flow cytometry using a DAPI staining. **C** cells were treated as in B using Mitomycin C (MMC, 200 µM) or Staurosporine (STS, 1 μM) in combination with the pan-caspase inhibitor qVD-OPH (20 μM), as a « CICD » stimulus for 36 h and cell death was analyzed by flow cytometry using a DAPI staining. **D** HeLa siCont and HeLa siUBR2 were treated either with UV (40 J/m^2^, as an apoptosis inducer) or with a combination of UV + qVD-OPH (20 μM) as a CICD stimulus. Cell death was measured in real time using PI staining and the Incucyte ZOOM Live-Cell Imaging system. **E** HeLa cells were transfected either with a non-targeting siRNA (siCont) or with three different siRNAs targeting UBR2 and were treated with Actinomycin D (1 µM) and qVD-OPH (20 μM) for the indicated times. Whole-cell lysate was analyzed for Smac expression by immunoblotting, Hsp60 was used as a loading control. **F** HeLa Smac-GFP cells were transfected with the indicated siRNA and were either not treated (NT), treated with Actinomycin D alone (1 μM) as an « apoptosis » stimulus or in combination with the pan-caspase inhibitor qVD-OPH (20 μM) as a « CICD » stimulus for 24 h. Representative FACS histogram showing the decrease of Smac expression after CICD treatment, as a read-out of MOMP. NT = not treated. **G** Quantification of Mean Fluorescence Intensity (MFI) of SMAC-GFP cells presented in F. Data are expressed as mean ± s.d (*n* = 3), immunoblots, FACS and incucyte measurements are representative of 3 or more individual experiments. **p < 0.01, ***p < 0.001, N.S: non-significant according to a two-way ANOVA.

To investigate if the UPS could be involved in CICD regulation, we first tested the effect of the general proteasome inhibitor Velcade (bortezomib) on apoptosis- and CICD-induced death. The Velcade dose used was effective as judged by the stabilization of the short-lived protein Mcl-1 (Fig. 3A). Importantly, in our settings, Velcade did not modulate Act-D-induced apoptosis while it sensitized HeLa cells towards CICD (induced by Act D + qVD-OPH, Fig. 3A), confirming the central role of the UPS in the control of this form of cell death.

To validate any potential new regulator of CICD, we performed a secondary screen by knocking down several UPS targets identified in the initial screen and by investigating their impact on CICD (Figure S2A–D). Importantly, to avoid any potential artefact (clone selection) that could be due to the use of HeLa-Apaf-1 KD cells that we generated for the initial screen, all the secondary screen and following experiments were realized using WT HeLa cells (or indicated cells) in presence of qVD-OPH and using newly generated siRNA.

Among the E3 ligases identified as potential CICD regulators (Fig. 2C), UBR2 (Ubiquitin Protein Ligase E3 Component N-Recognin 2) stood out as the best candidate. UBR2 is a RING E3 ligase that belongs to the N-End rule pathway, is implicated in spermatogenesis and can be up-regulated during cancer and cachexia, promoting tumor growth and metastasis^14–16^. UBR2 downregulation using a novel set of three independent siRNA (Figure Sup 3A) did not sensitize HeLa cells towards Act D-induced caspase-dependent apoptosis (Figure Sup 3B) but sensitized cells towards CICD (induced by the combination of ActD and qVD-OPH), as shown in Fig. 3B. We then extended our observation by showing that UBR2 KD cells are sensitized to CICD regardless of the stimuli used: mitomycin C (DNA cross linker), staurosporine (pan kinase inhibitor) or UV irradiation (DNA damaging agent, Fig. 3C–D) or MOMP inducers (using the BH3 mimetic ABT-737, Figure Sup 3C) but did not sensitize cells towards apoptosis induced by the same agents (Figure Sup 3 B and C).

As previously mentioned, MOMP is a critical step in CICD regulation, we therefore verified if UBR2 knock-down could impede it by looking at Smac degradation once release from mitochondria. We observed no difference in Smac degradation between control cells and cells KD for UBR2 (Fig. 3E). With the aim of confirming this result, we took advantage of HeLa cells expressing Smac fused with GFP protein^17^ in which the decrease in GFP fluorescence is a readout of MOMP. We confirmed that even though UBR2 knock-down was sensitizing cells to CICD, it was not impinging on MOMP but seems to act rather downstream of it (Fig. 3F–G).

Thus, we concluded that the E3 ligase UBR2 is a potential new regulator of CICD that does not impact on MOMP nor on apoptosis.

### UBR2 regulates CICD but no other main non-apoptotic forms of death (necroptosis, ferroptosis, or autophagy-related)

We established that UBR2 could regulate CICD but not apoptosis. We then wondered if UBR2 could regulate the main characterized forms of non-apoptotic death: necroptosis, ferroptosis, or autophagic cell death^1^.

We first investigated the potential role of autophagy in our model. To this end, we used HeLa cells invalidated for a main autophagy regulator ATG12 using CRSIP/Cas9 technology^18^. We recently characterized those cells^19^. UBR2 knock-down could sensitize cells to caspase-independent cell death whether ATG12 was present or not (Sup Fig. 4A), suggesting that autophagy is not involved in UBR2-mediated regulation of CICD and that UBR2 cannot control autophagic cell death.

Since HeLa cells are not able to die by necroptosis as RIPK3 is absent^20^, it was unlikely that UBR2 could sensitize cells toward this form of death. However, to fully address this question, we used 3T3-SA cells that are a classical cellular model to study necroptosis upon stimulation with TNFα in presence of the caspase inhibitor zVAD.fmk^21^. While 3T3-SA cells died by necroptosis upon this co-treatment (as judged by the ability of Nec-1 to prevent it), UBR2 knock-down did not sensitized cells towards this form of regulated cell death (Sup Fig. 4B–C).

Using the same rational, we treated WT HeLa or HeLa cells knock-down for UBR2 with Erastin (a ferroptosis inducer). While this form of death was efficiently prevented by Ferrostatin-1 as expected^22^, UBR2 KD did not sensitize the cells to ferroptosis (Sup Fig. 4D).

Overall, these data indicate that UBR2 is a regulator of CICD and not of apoptosis, autophagic cell death, necroptosis or ferroptosis.

### UBR2 overexpression protects cell from CICD but not from apoptosis

**Fig. 4:**
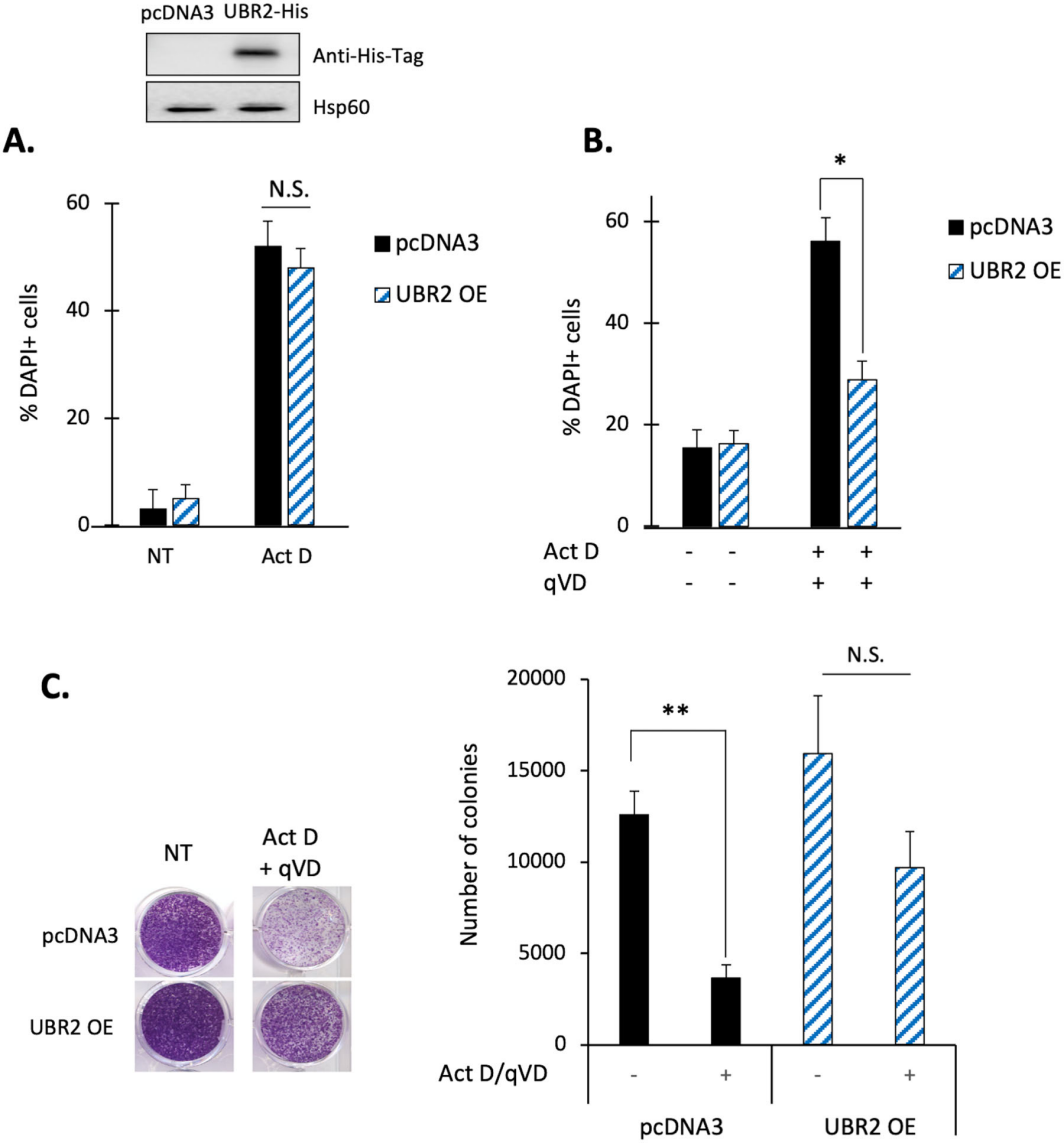
UBR2 overexpression protects cells from CICD but not from apoptosis. **A** HeLa cells were transfected to transiently overexpress (OE) the control vector (pcDNA_3_) or UBR2-His-tagged and then cells were either not treated (N.T) or treated with Actinomycin D (1 μM) for 8 h as an « apoptosis » stimulus. Cell death was measured by flow cytometry using a DAPI staining. Whole-cell lysates were analyzed for UBR2 over-expression by immunoblotting against His-tag, Hsp60 was used as a loading control. **B** as in **A** but cells were either untreated or treated with Act D (1 µM) and qVD-OPH (20 μM) for 48 h to induce CICD. **C** Clonogenic assay of pcDNA3 or UBR2-His-tagged overexpressing HeLa cells treated were treated with Actinomycin D (1 µM) and qVD-OPH (20 μM) for 24 h. Pictures were taken 10 days following the treatment and quantified (right panel). NT = not treated. Data are expressed as mean ± s.d (*n* = 3). *p < 0.05, **p < 0.01, N.S: non-significant according to a two-way ANOVA.

We previously showed that UBR2 knock-down could sensitize cells to CICD. We then wondered if, on the opposite, overexpression of UBR2 could protect cells from CICD? We, therefore, transfected HeLa cells with either an empty control vector (pcDNA3) or with a vector encoding for UBR2-His-tagged and stimulated cells with either Act D alone (apoptosis stimulus) or in combination with qVD-OPH (CICD stimulus). Interestingly, UBR2 overexpression did not modulate apoptosis (Fig. 4A) while it specifically reduced the number of dead cells under CICD treatment, compared to pcDNA3 transfected cells (Fig. 4B). To analyze if UBR2 overexpression was delaying CICD or protecting cells from it, we performed a clonogenic test. For that matter, HeLa WT or cells overexpressing UBR2 were left untreated (NT) or treated with Act D with qVD-OPH (CICD conditions) and we monitored the ability of the cells to form clones. While CICD could efficiently limit colony formation of control cells, UBR2 overexpressing cells could resist CICD treatment, recover and grow to form clones (Fig. 4C), suggesting that overexpression of UBR2 is sufficient to protect some cells from CICD and to allow clonogenic expansion.

### UBR2 prevents CICD through the control of the MAPK/Erk pathway

**Fig. 5:**
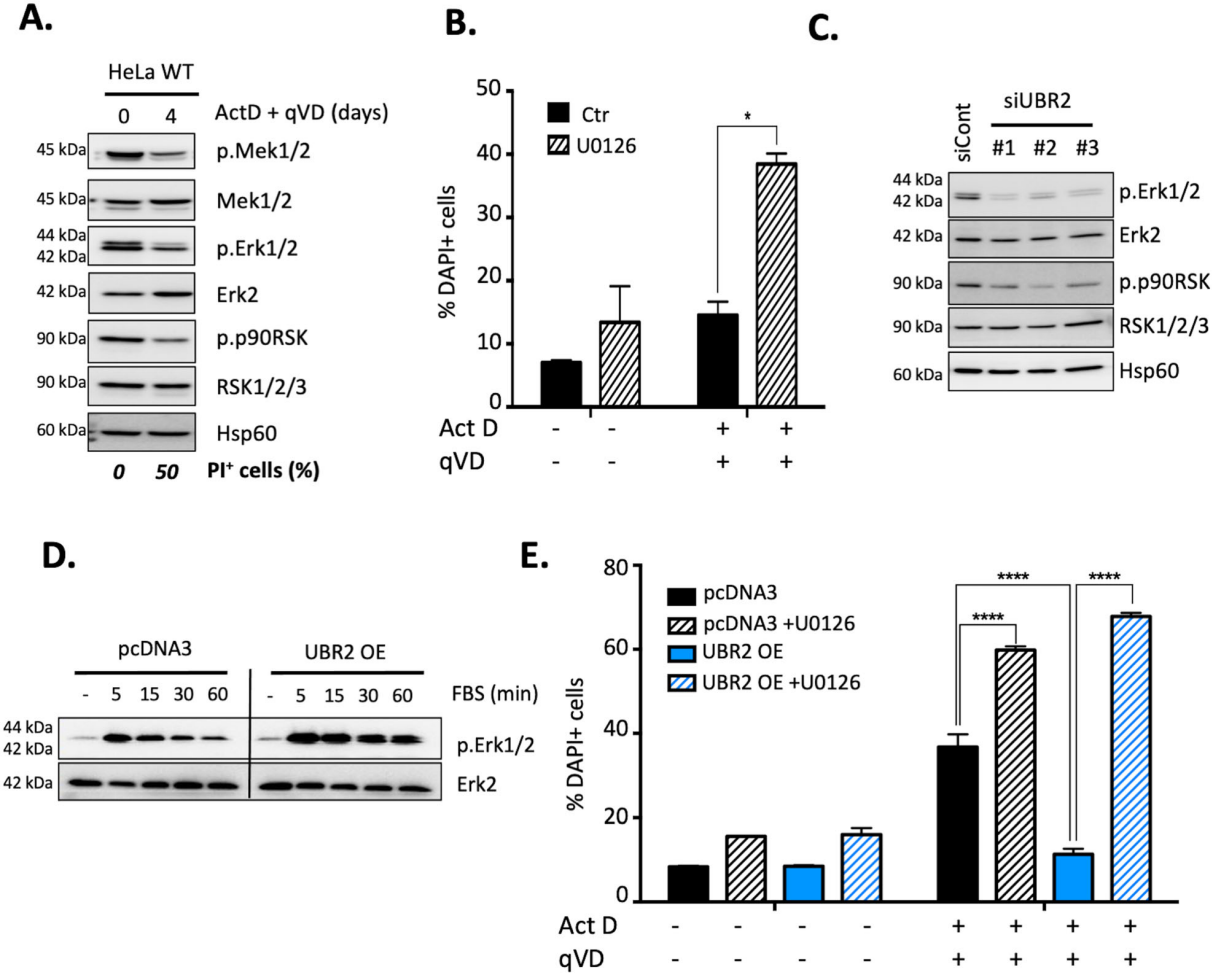
UBR2 protection towards CICD depends on MAPK/Erk signaling pathway. **A** HeLa cells were treated with Actinomycin D (1 µM) and qVD-OPH (20 μM) for 4 days and the MAPK/Erk pathway was assessed by immunoblots. Activation was assessed by looking at the phosphorylation state of Mek1/2, Erk1/2, and p90Rsk. Hsp60 was used as a loading control. % of PI positive cells are indicated below the immunoblots. **B** HeLa cells were treated as previously or in combination with the MEK1/2 inhibitor U0126 (10 μM) for 24 h. Cell death was measured by flow cytometry using a DAPI staining. **C** HeLa cells were transfected with the three independent siRNA targeting UBR2. 48 h later, whole-cell lysates were analyzed for p.Erk1/2, Erk1/2, p.p90RSK, and RSK1/2/3 by immunoblotting. Hsp60 was used as a loading control. **D** HeLa cells were transfected to transiently overexpress the control vector (pcDNA_3_) or UBR2-His-tagged. After 24 h, cells were cultivated without serum for 16 h and the next day were stimulated with 10% FBS for the indicated times. Whole cell lysates were analyzed for p.Erk1/2 and Erk2 (used as a loading control). **E** HeLa cells were transfected to transiently overexpress (OE) the control vector (pcDNA_3_) or UBR2-his-tagged and were treated with Actinomycin D (1 µM) + qVD-OPH (20 μM) alone or in combination with the MEK1/2 inhibitor U0126 (10 μM) for 3 days. Cell death was measured by flow cytometry using a DAPI staining. Data are expressed as mean ± s.d (*n* = 3) immunoblots are representative of 3 individual experiments. *p < 0.05, ****p < 0.0001, N.S: non-significant according to a two-way ANOVA.

As UBR2 has previously been linked to the survival MAP Kinases signaling pathway^15^, we first monitored the status of this pathway during a CICD time course in HeLa cells (Fig. 5A). During CICD, MAPK/Erk pathway was decreased over time as judged by the phosphorylation state of the proteins MEK1/2, Erk1/2 and p90RSK (Fig. 5A). Moreover, when MAPK signaling was inhibited upon treatment with U0126 (MEK1/2 inhibitor), cells were more sensitive toward CICD compared to control cells (Fig. 5B). Interestingly, using three independent siRNA targeting UBR2, we could show that MAPK signaling pathway is inhibited in UBR2 knock-down HeLa cells, as there was a dramatic decrease in the phosphorylation of ERK1/2 and p90RSK (Fig. 5C). We also validated that modulation of UBR2 is impacting on the activation of MAPK/Erk pathway not only in HeLa cells but also in MDA-MB-231 breast cancer cells (Sup Fig. 5).

On the opposite, cells overexpressing UBR2 could activate more efficiently and for a longer time the MAPK/Erk pathway than pcDNA3 control cells in response to a triggering stimulus (Fig. 5D). Altogether, these data led us to wonder if the modulation of the MAPK/Erk could be involved in UBR2-mediated protection from CICD. As shown in Fig. 5B, U0126 treatment can increase CICD in control cells (pcDNA3), more strikingly, it can completely abolish UBR2-dependent protection from CICD (Fig. 5E).

Collectively, these data suggest that UBR2 can protect cancer cells from CICD through the activation of the prosurvival MAPK/Erk signaling pathway.

### UBR2 is overexpressed in cancer cells and contributes to their protection from CICD

**Fig. 6:**
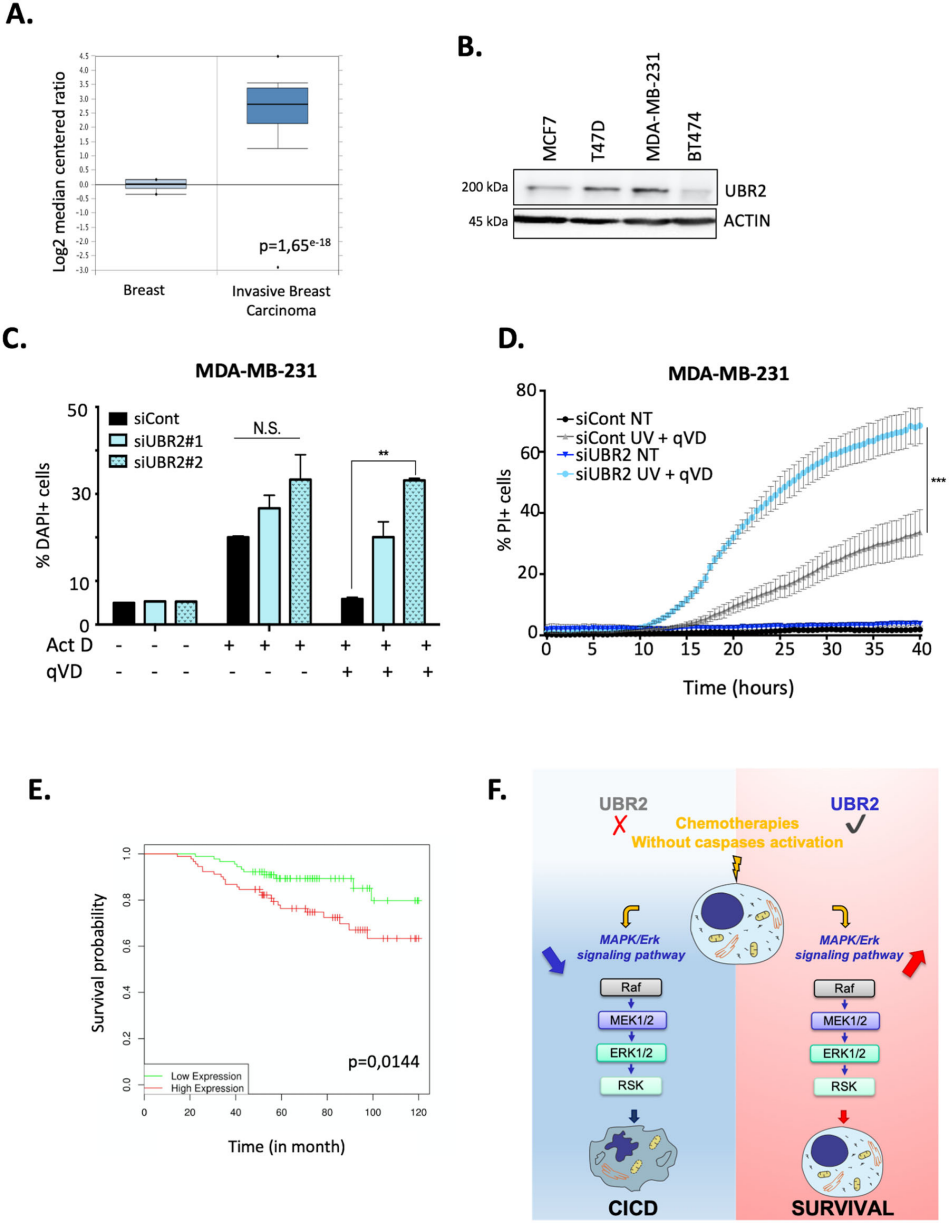
UBR2 is widely expressed in cancer cells and contributes to their protection from CICD. **A** UBR2 mRNA expression between healthy tissue (breast) and cancer tissue (invasive breast carcinoma). Data were obtained from the Oncomine database. **B** Immunoblots of UBR2 expression in the indicated breast cancer cell lines. Actin was used as a loading control. **C** MDA-MB-231 cells were transfected with the indicated siRNA and treated with Actinomycin D alone (1 μM) as an « apoptosis » stimulus or in combination with the pan-caspase inhibitor qVD-OPH (20 μM) as a « CICD » stimulus for 24 h. Cell death was measured by flow cytometry using a DAPI staining. Data are expressed as mean ± s.d (*n* = 3). **D** Real-time quantification of MDA-MB-231 cells transfected with the indicated siRNA and stained for Propidium Iodide in response to treatment with UV (40 J/m^2^) in combination with qVD-OPH (20 μM) as a CICD stimulus, using the Incucyte ZOOM Live-Cell Imaging system. NT = not treated. **E** DRUGSURV Database shows that High levels of UBR2 mRNA (*n* = 91) predicts a poor survival compared to low levels (*n* = 90) in patients suffering from breast cancer. **F** graphic summary of our work, see text for details. **p < 0.01, ***p < 0.001 according to a two-way ANOVA. N.S: non-significant. Data are expressed as mean ± s.d (*n* = 3) immunoblots and incucyte data are representative of 3 individual experiments.

One characteristic of cancer cells is their ability to escape from several types of cell death including apoptosis and CICD. We determined UBR2 expression levels in human cancers. We observed that UBR2 is widely expressed among different human cancer tissues, especially in lymphomas, prostate, and breast cancer (Figure S6). The most dramatic difference observed between normal and cancer human tissue was found in healthy breast versus invasive breast carcinomas (Fig. 6A). We observed that independent breast cancer cell lines presented variable amount of UBR2 protein expression, with the highest expression in MDA-MB-231 breast cancer cells (Fig. 6B). Therefore, we first determined the sensitivity of those cells to CICD (using a combination of ActD and qVD). Strikingly, MDA-MB-231 cells presented a massive resistance toward CICD while they were sensitive towards apoptosis (Fig. 6C). This result was confirmed using another CICD triggering agent (UV + qVD-OPH) using real-time analysis of cell death (Incucyte technology, Fig. 6D). Interestingly, UBR2 knock-down breast cancer cells are more sensitive to CICD than control cells, suggesting that overexpression of UBR2 in MDA-MB-231 cells could participate to the escape from CICD. Collectively, these data suggest that UBR2 is overexpressed in cancer cells, especially in breast cancer and that it could participate to CICD resistance. This is supported by the observation that breast cancer patients with higher UBR2 expression have a poor survival compared to patients with low UBR2 expression upon treatment (Fig. 6E).

## Discussion

Regulation of programmed cell death plays a central role in numerous physiological and pathological processes, such as cancers. Over the last decades, we made tremendous progress in understanding how cell deaths are regulated, however we have also come to realize that those complexes, and likely intertwined processes, are still only partially understood^1^. Most stimuli, including chemotherapeutic treatments (such as Act D, etoposide, paclitaxel, or more recently BH3 mimetics), initiate apoptosis through the intrinsic pathway by engaging or sensitizing cells to MOMP. In conditions where caspase cannot be activated, MOMP will very frequently still lead to cell death, defined by the generic name of CICD. It has been extensively described that CICD is a slow, compared to apoptosis, but a very efficient way to kill tumor cells, suggesting that inducing tumor-specific CICD may represent a new and efficient way to limit tumor growth. So far, the only well-characterized inhibitor of CICD is the glycolytic enzyme GAPDH^11,23,24^. However, as GAPDH is a central metabolic gene, targeting it may be challenging as a therapeutic option. Here we identified that UBR2 is a novel regulator of CICD in an MAPK/Erk dependent manner (Fig. 6F).

In this study, we performed a siRNA genome-wide screen to identify novel regulators of CICD using a high throughput imaging screening system. siRNAs were transfected 24 h prior to the stimulation with Act D for 72 h, condition resulting in approximatively 50% dead cells in the control condition. Due to technical consideration, we did not incubate siRNA for a longer period of time. We therefore cannot exclude that our screen would not be able to identify any long-lived proteins that would be minimally reduced in our settings. Among the 1,037 target genes identified as potential regulators of CICD, bioinformatics analysis has revealed that a large proportion of hits are involved in ribosomal function. While we did not study this further, it is very likely to be linked to the use of Act D as a cell death inducer in our screen. Interestingly, our genome-wide screening identified nearly twenty genes involved at each stage of the ubiquitin-proteasome pathway (UPS), including ubiquitins, ubiquitin transfer (E2), and binding (E3) enzymes as well as proteasome subunits. Proteasome inhibition through the use of Velcade (bortezomib) sensitizes cells to CICD but not towards Act D-induced apoptosis and therefore suggested further the involvement of UPS in CICD’s control (Fig. 3A). Interestingly, the UPS has already been shown to be involved in the regulation of non-apoptotic death in *C.elegans* where the linker cell, specific to the male gonad, dies during the development of the nematode by a non-apoptotic process governed by the protein HSF-1. Genetic and functional studies suggest that HSF-1 could work by activating components of the UPS^25^.

Among UPS genes identified in our screen, we find four E3 ubiquitin ligases (EDD, ITCH, ARIH1, and UBR2), enzymes that carry the specificity of the reaction of the UPS. Since during the validation phase knock-downs of ITCH and EDD had no effect on the regulation of CICD (Figure S2), we excluded these 2 genes from potential regulatory proteins. Mitophagy has been shown previously to be an established defense process by cancer cells to resist CICD^11^. We recently established that ARIH1 was a key regulator of mitophagy in cancer cells and that its knock-down could sensitize cells towards chemotherapy-induced apoptosis^19^. In line with those results, we observed that ARIH1 knock-down sensitizes cells to cell death in the presence or absence of caspase activation (i.e., upon induction of apoptosis or CICD, (Figure S2E).

Using a wide variety of techniques, we established that UBR2 knock-down sensitize cells towards CICD but not towards apoptosis regardless of the stimuli used. Also, we showed that overexpression of UBR2 protects cells against CICD (Fig. 4) and that UBR2 is found overexpressed in many types of cancer (Figure S6 and Fig. 6) including breast cancers. It appears that UBR2 is more expressed in grade 2 breast cancer patients but is not associated with the age of the patient. Interestingly, genome-wide screening in triple-negative breast cancer cells revealed that these cells were highly dependent on the proteasome and that this dependence could be exploited as a vulnerability to induce death cells using proteasome inhibitor^26^.

A key remaining question is how UBR2 can control the Erk/MAPK pathway to prevent CICD? UBR2 is part of the “N-End Rule” pathway which allows a proteasomal degradation of proteins with an N-terminal destabilizing part^27^. Therefore, we could hypothesize that UBR2 specifically ubiquitinates a negative regulator of the Erk/MAPK pathway, leading to its degradation by the proteasome, thereby facilitating the activation of the Erk / MAPK pathway. However, such substrate remains to be identified.

Cell death is often seen as an endpoint, however we should keep in mind that in vivo the way a cell is dying will directly impact on the immune response^28^. Several forms of death will not stimulate an immune response (which will benefit the organism in “normal” condition), however, in response to a chemotherapeutic agent, the patient would benefit if the dying cancer cells could stimulate and induce an efficient anti-cancer immune response. How a dying cell becomes immunogenic is still unclear and highly debated^28^. Nevertheless, it was recently established that upon caspase-inhibition, CICD could alert the immune system in a type I interferon (IFN) response and nuclear factor kappa-light-chain-enhancer of activated B cells (NF-κB) dependent manner^8^. Interestingly, it was recently suggested that UBR2 could mediate NLRP1B (NLR Family Pyrin Domain Containing 1) inflammasome induction^29^, we could therefore speculate that UBR2-dependent control of CICD may impact on the immunogenicity of the cancer cell, at least in part, through cytokines production of the dying cells, however, this point will be further investigated later.

In conclusion, it has been reported that caspase activation may have advert effects as it could enhance tumoral aggressiveness^30^. Here we are identifying a novel and specific regulator of CICD which inhibition could enhance this type of death, therefore providing novel therapeutic options.

## Supplementary information

Supplementary Figure Legends

Figure S1

Figure S2

Figure S3

Figure S4

Figure S5

Figure S6

Supplemental Table 1
