# Supplementary material for: The E3 ligase UBR2 regulates cell death under caspase deficiency via Erk/MAPK pathway
Source: Cell Death Dis. 2020 Dec 8;11(12):1041. doi: 10.1038/s41419-020-03258-3 (PMC7721896; doi:10.1038/s41419-020-03258-3)
Supplement: Supplementary file 1 — Supplementary Figure Legends [file 41419_2020_3258_MOESM1_ESM.docx]

**Supplemental Information**

**Supplemental Figure legends:**

**Figure S1: Illustration of Genome wide screening**

A) Hela-shAPAF1 cells were treated with Actinomycin D (1 μM final) for 72h and were stained with CMFDA (10 µM) and Hoechst 33342 (2,5 μg/mL). Images were the acquired on the Operatta at 10X magnification (Brightfield, Alexa 488 and DAPI channels). ROI selection according to the Cell roundness/contrast parameter shows that dying cells are correctly identified (red), while almost all flat, attached cells were not selected (green).

B) GO analysis of molecular functional or biological process for the sensitizing versus the CICD protector genes identified in the pan genomic screen.

**Figure S2: secondary validation of some of the UPS hits identified.**

**A) B)** **C)** and **D)** HeLa cells were transfected with the indicated siRNA [20nM] for 48 hours and were treated with Actinomycin D (1 µM) and qVD-OPH (20 μM) for 24 hours and cell death was measured by flow cytometry using a Propidium Iodide staining. Each siRNA transfection was verified by immunoblotting using specific antibody. Erk2 or Hsp60 were used as loading controls.

Data are expressed as mean ± s.d (n=3) immunoblots are representative of 3 individual experiments.

**Figure S3: knock down of UBR2 does not sensitizes cells to apoptosis-induced death**

**A)** HeLa cells were transfected with a control of a siRNA targeting UBR2 [20nM]. UBR2 expression was then determined by immunoblots 48 hours later. Actin is used as a loading control.

**B)** HeLa cells were transfected with indicated siRNA and 48 hours later treated with Act D (1µM), Mitomycin C (MMC, 200 µM) or Staurosporine (STS, 1 μM) for 6 hours. Cell death was measured by FACS using DAPI staining.

**C)** HeLa cells were transfected with the indicated siRNA for 48 hours and then treated with 100 µM ABT-737 for 4 hours or with ABT-737 (100 µM) + q-VD-OPH (20 µM) for 4 days. Cell death was measured by FACS analysis using DAPI staining.

Data are expressed as mean ± s.d (n=3) immunoblots ** p< 0.01, N.S. non-significant.

**Figure S4: UBR2 regulates CICD but no other non-apoptotic forms of death (necroptosis, ferroptosis or autophagy related)**

**A)** HeLa control cells (crispr CTL) and HeLa cells invalidated for ATG12 (crispr ATG12) were transfected with the indicated siRNA and were treated 48 hours later with Actinomycin D alone (1 μM) as an « apoptosis » stimulus or in combination with the pan-caspase inhibitor qVD-OPH (20 μM) as a « CICD » stimulus for 24 hours. Cell death was measured by flow cytometry using a DAPI staining.

**B)** 3T3-SA cells were transfected with either a siRNA control or with two different siRNA targeting the mouse isoform of UBR2. 48 hours later cells were treated with TNFα and zVAD.fmk alone (10 ng/mL, 25 μM) for induction of necroptosis or in combination with Necrostatin-1 (30 μM) for 24 hours. Cell death was measured by flow cytometry using a DAPI staining.

**C)** Whole cell lysates of 3T3-SA cells presented in B. were analyzed for UBR2 expression by immunoblotting. Actin was used as a loading control.

**D)** HeLa cells were transfected with the indicated siRNA and 48 hours later were treated with Erastin alone (20 μM) as a ferroptosis stimulus or in combination with Ferrostatin-1 (Fer-1, 1 μM, a ferroptosis inhibitor) for 24 hours. Cell death was measured by flow cytometry using a DAPI staining.

Data are expressed as mean ± s.d (n=3) immunoblots are representative of 3 or more individual experiments.

***p<0.001 according to a two-way ANOVA. N.S: non-significant.

**Figure S5: UBR2 controls MAPK/Erk signaling pathway in MDA-MB-231 cells**

MDA-MB-231 cells were transfected with a non-targeting siRNA (siCont) or a siRNA targeting UBR2 [20 nM] for 48 hours and were treated with Actinomycin D (1 µM) and qVD-OPH (20 μM) for the indicated time. Whole cell lysates were analyzed for p.Erk1/2, Erk1/2, p.p90RSK and RSK1/2/3 by immunoblotting. Hsp60 was used as a loading control.

Immunoblots are representative of 3 or more individual experiments.

**Figure S6 : UBR2 mRNA expression in indicated human cancers.**

Data were obtained from Oncomine website.

**supplementary table 1: List of candidates’ genes for CICD regulation**

Table of Sensitizer and Protector genes identified from the Genome siRNA lethality screen. In red, are the gene belonging to the Ubiquitin Proteasome pathway.
