# Supplementary figures and images for: The E3 ligase UBR2 regulates cell death under caspase deficiency via Erk/MAPK pathway
Source: Cell Death Dis. 2020 Dec 8;11(12):1041. doi: 10.1038/s41419-020-03258-3 (PMC7721896; doi:10.1038/s41419-020-03258-3)

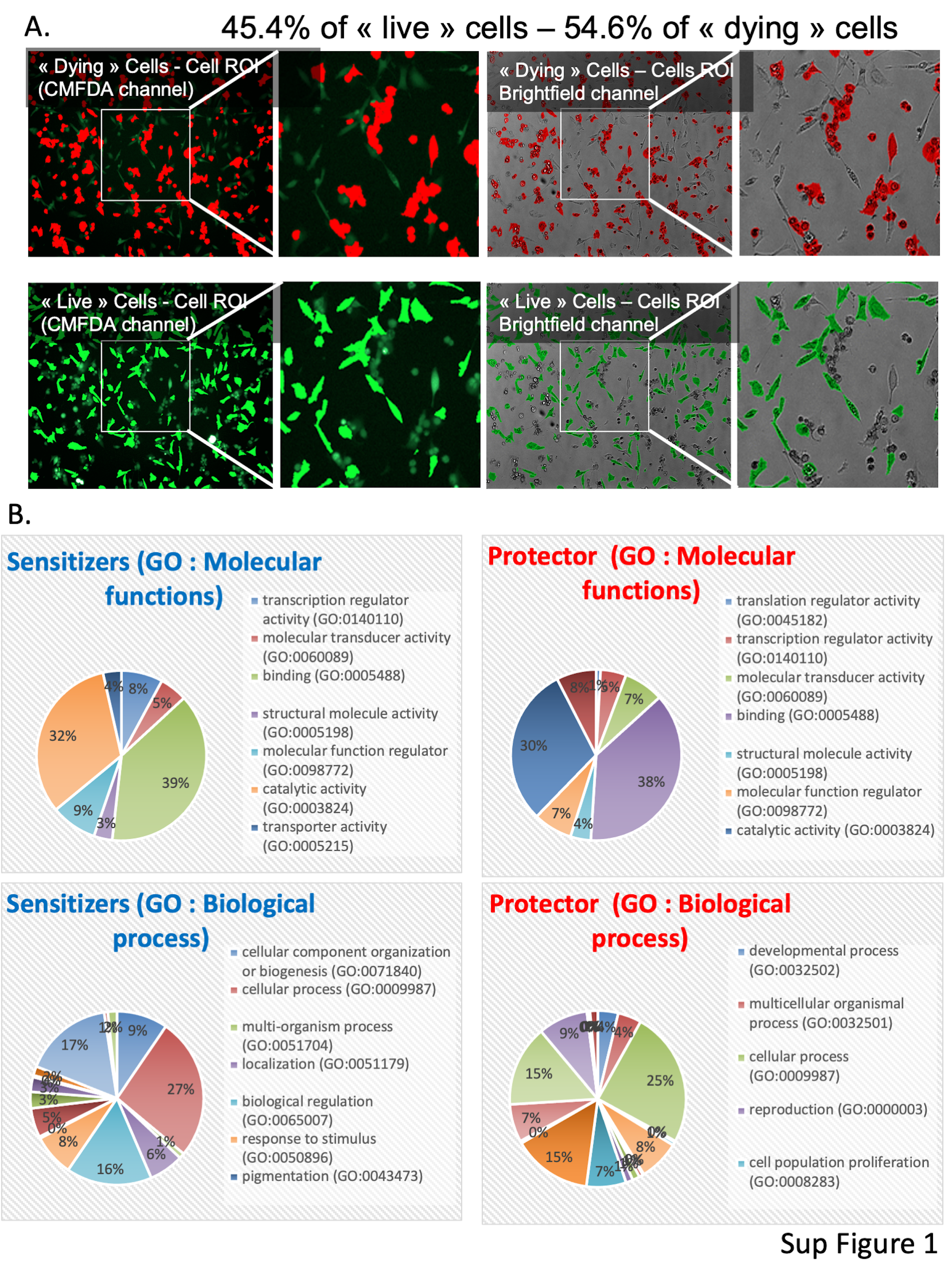

Supplement: Supplementary file 2 — Figure S1 [file 41419_2020_3258_MOESM2_ESM.png]

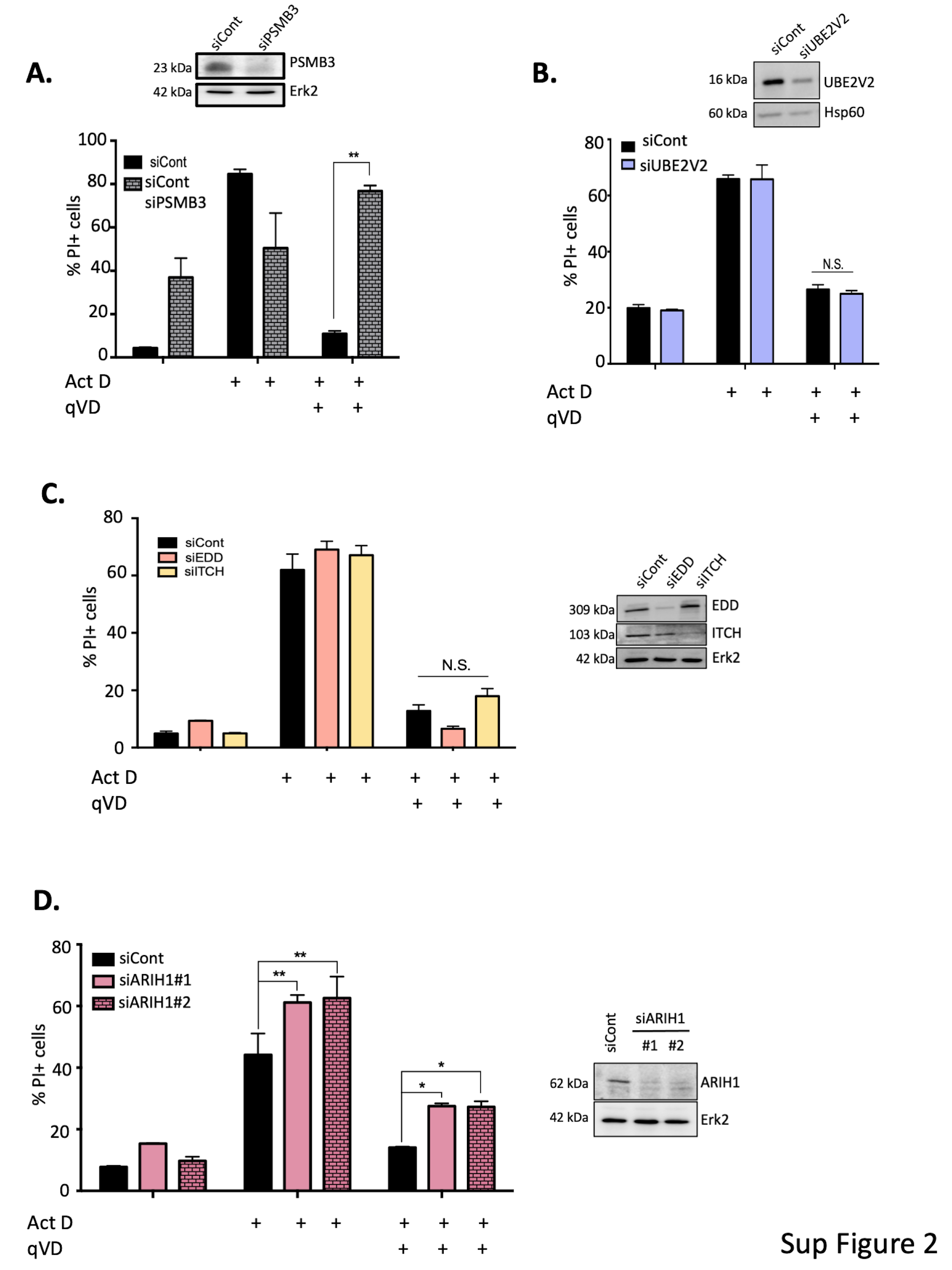

Supplement: Supplementary file 3 — Figure S2 [file 41419_2020_3258_MOESM3_ESM.png]

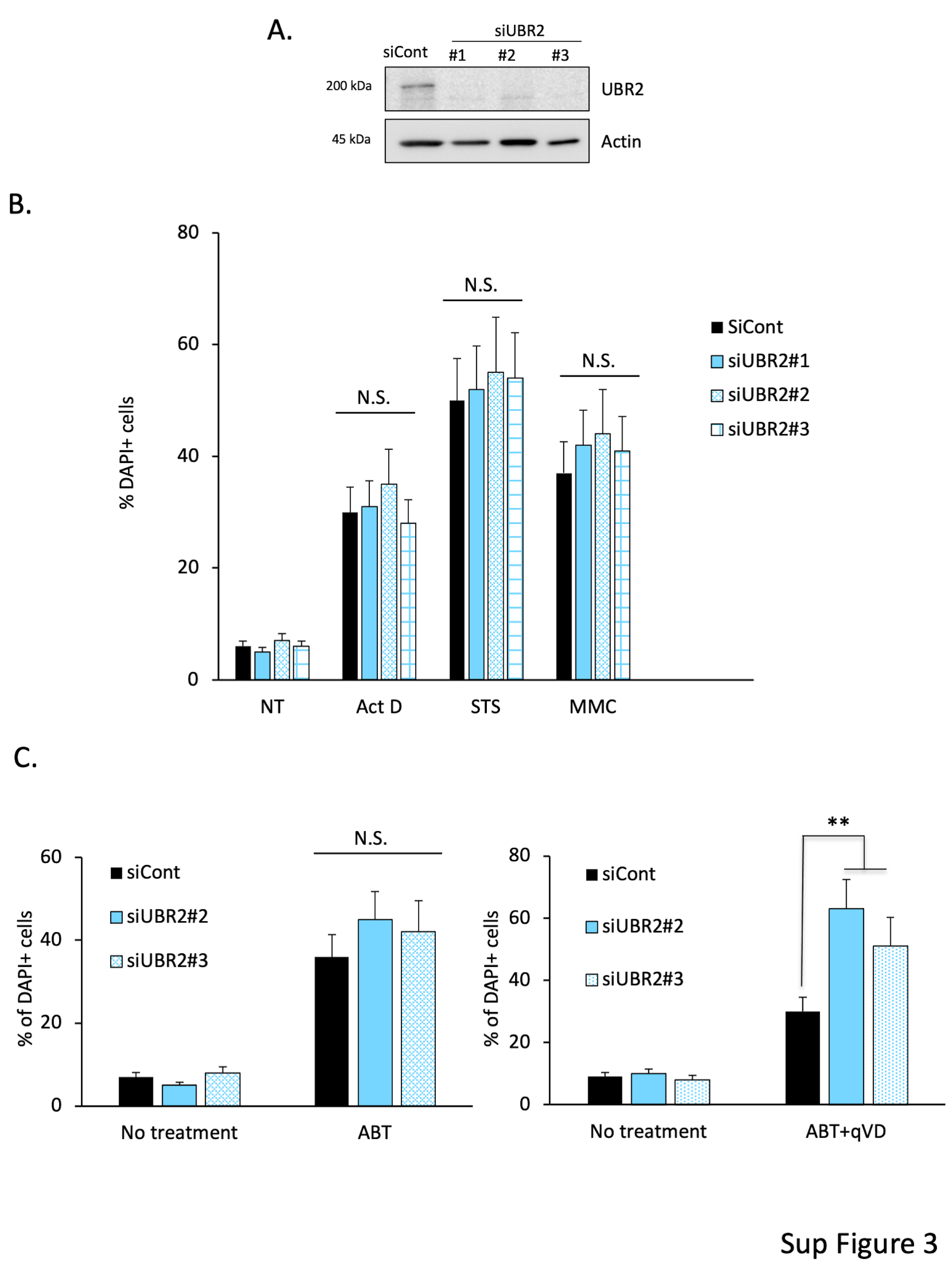

Supplement: Supplementary file 4 — Figure S3 [file 41419_2020_3258_MOESM4_ESM.png]

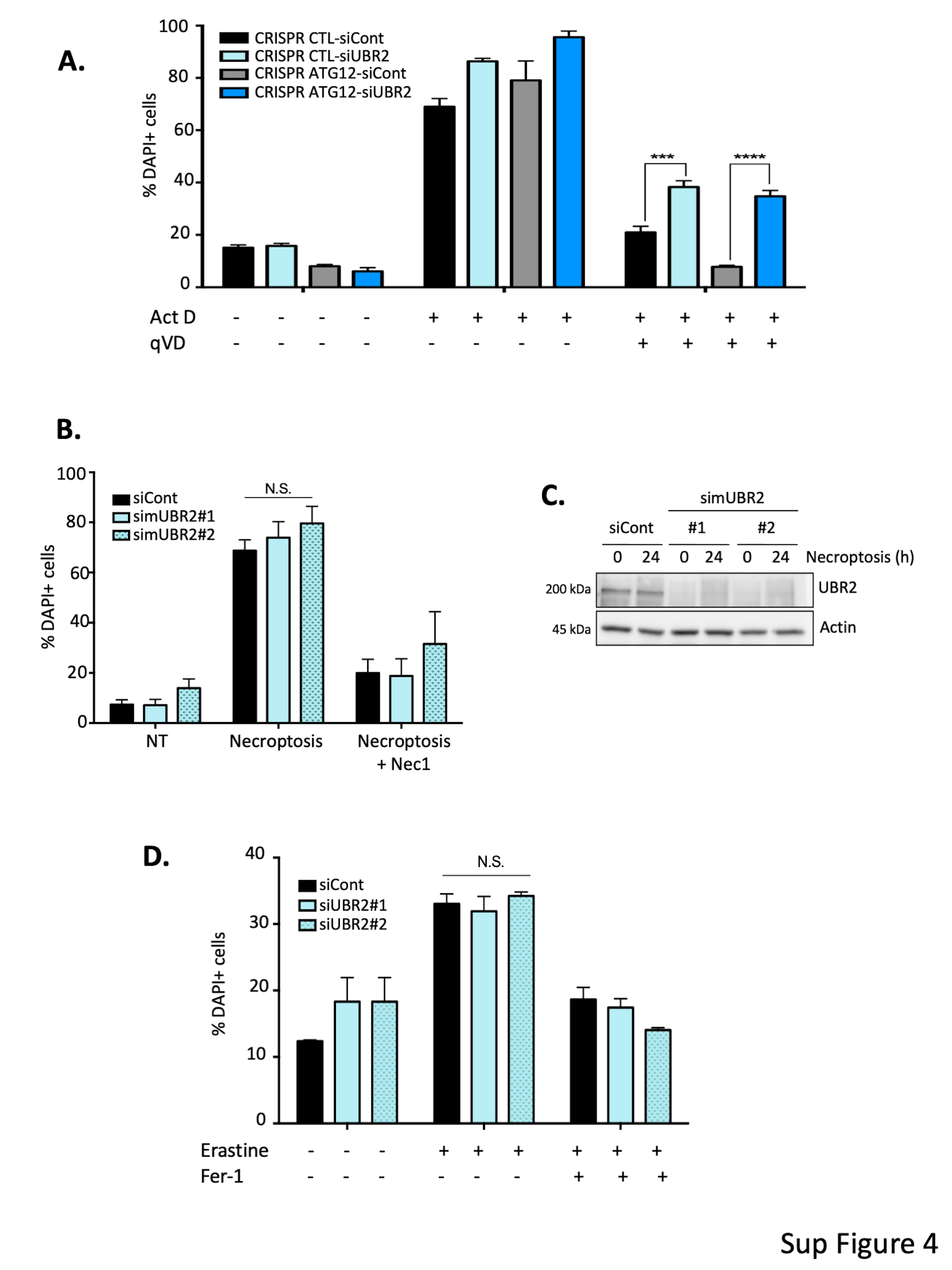

Supplement: Supplementary file 5 — Figure S4 [file 41419_2020_3258_MOESM5_ESM.png]

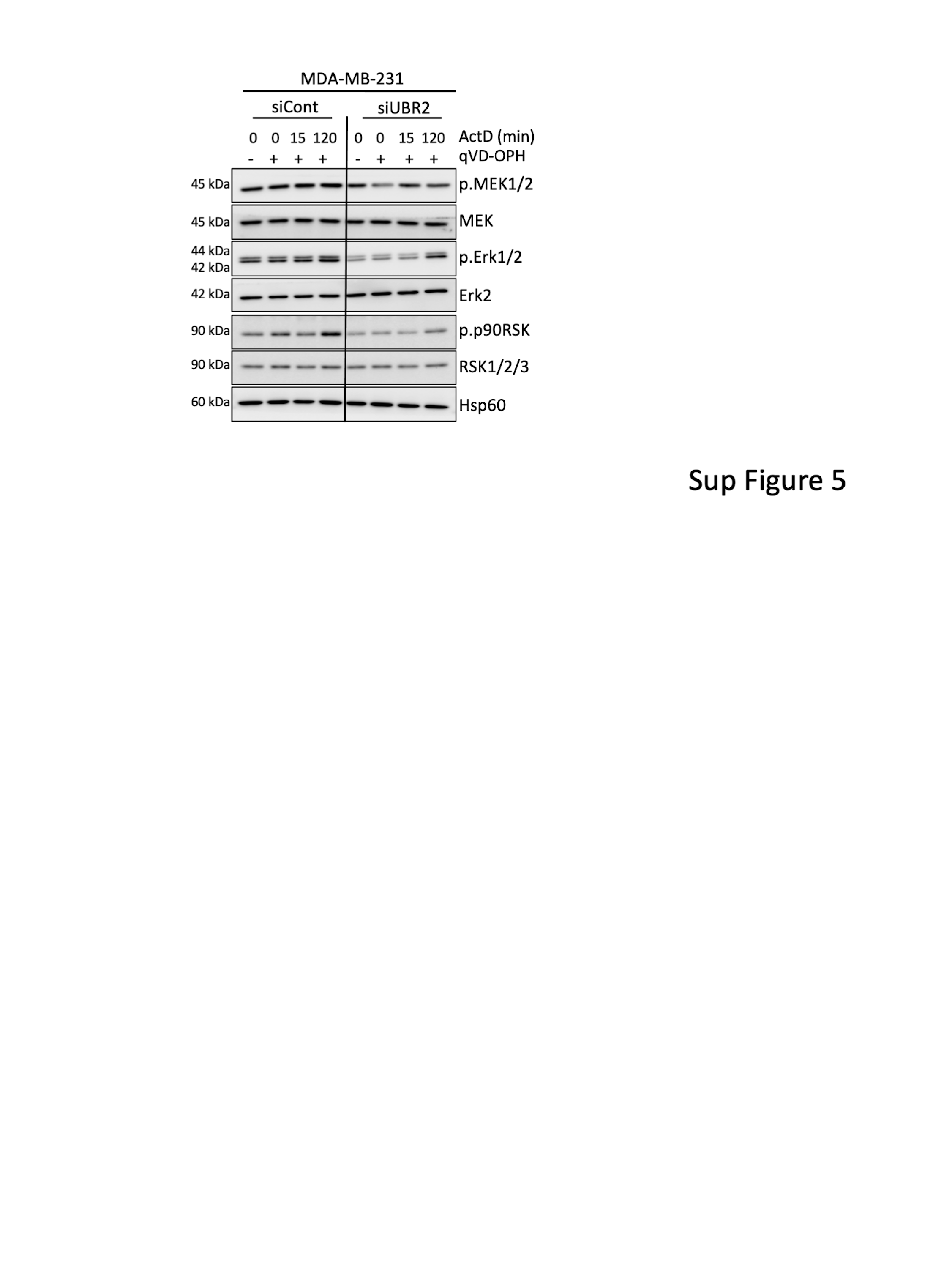

Supplement: Supplementary file 6 — Figure S5 [file 41419_2020_3258_MOESM6_ESM.png]

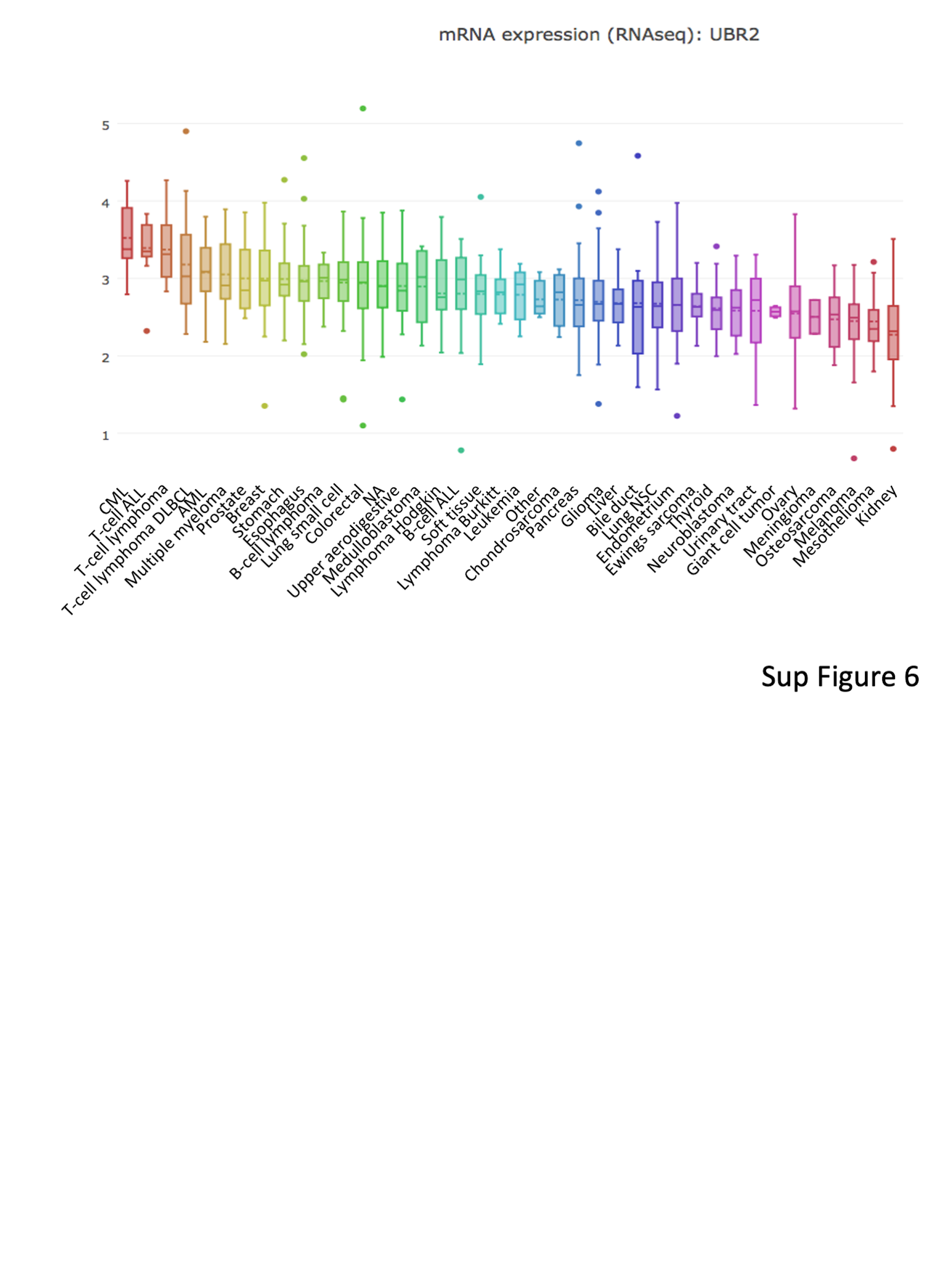

Supplement: Supplementary file 7 — Figure S6 [file 41419_2020_3258_MOESM7_ESM.png]
